# Density and temperature characterization of long-scale length, near-critical density controlled plasma produced from ultra-low density plastic foam

**DOI:** 10.1038/srep21495

**Published:** 2016-02-29

**Authors:** S. N. Chen, T. Iwawaki, K. Morita, P. Antici, S. D. Baton, F. Filippi, H. Habara, M. Nakatsutsumi, P. Nicolaï, W. Nazarov, C. Rousseaux, M. Starodubstev, K. A. Tanaka, J. Fuchs

**Affiliations:** 1 LULI - CNRS, Ecole Polytechnique, CEA: Universit´e Paris-Saclay; UPMC Univ Paris 06: Sorbonne Universites - F-91128, Palaiseau cedex, France; 2 Institute of Applied Physics, 46 Ulyanov Street, 603950 Nizhny Novgorod, Russia; 3 Graduate School of Engineering, Osaka University, Japan; 4 La SAPIENZA, University of Rome, Dip. SBAI, 00161 Rome, Italy; 5 European X-Ray Free-Electron Laser Facility (XFEL) GmbH, Hamburg, Germany; 6 Univ. Bordeaux - CEA - CNRS, CELIA, UMR 5107, F-33405 Talence, France; 7 University of St Andrews, High Energy Laser Materials Laboratory, Unit 4, NTC, North Haugh, St Andrews, KY16 9SR, UK; 8 CEA, DAM, DIF, F-91297 Arpajon, France

## Abstract

The ability to produce long-scale length (i.e. millimeter scale-length), homogeneous plasmas is of interest in studying a wide range of fundamental plasma processes. We present here a validated experimental platform to create and diagnose uniform plasmas with a density close or above the critical density. The target consists of a polyimide tube filled with an ultra low-density plastic foam where it was heated by x-rays, produced by a long pulse laser irradiating a copper foil placed at one end of the tube. The density and temperature of the ionized foam was retrieved by using x-ray radiography and proton radiography was used to verify the uniformity of the plasma. Plasma temperatures of 5–10 eV and densities around 10^21^ cm^−3^ are measured. This well-characterized platform of uniform density and temperature plasma is of interest for experiments using large-scale laser platforms conducting High Energy Density Physics investigations.

Producing long-scale length, uniform density plasmas has been a long-desired goal of the plasma physics community in order to study a wide range of fundamental processes related to electromagnetic waves or particles with plasmas while eliminating the effects of plasma gradients which can, for example, modify the growth of induced instabilities^1^. There are various ways to achieve this in the regime of low-density plasmas, i.e. densities <10^20^ particles.cm^−3^: gas-plasma discharges^2^, high-pressure gas bags^3^, gas-filled capillaries^4^, gas-filled hohlraum^5^, or XFELs^6^ and particle beams^7^. In most of these configurations, the plasma is confined in order to keep a hot plasma in the desired state long enough for the investigation. Additionally, supplementary confinement methods (e.g. magnetic) can be applied.

However, reaching higher plasma densities, intermediate between those accessible with the above-mentioned platforms and the solid density has proved to be more difficult. Of particular interest is the production of such plasmas having density close or higher than the critical density for high-power laser beams (n_c_[cm^−3^] = 10^21^/(λ[μm])^2^ where λ is the laser wavelength). Doing so will indeed allow us to investigate e.g. laser-based ion acceleration^8^,^9^, or the relativistic or so-called “superpenetration” mode of laser propagation^10^ in such high density plasmas, but also ion^11^ or electron^12^ propagation and stopping in such dense plasma.

For a long time, exploded solid-density foils, which have large gradients, were used for such studies^10^ in high-density plasma. Currently, alternative high-pressure gas jets also allow the attainment of high densities^11^,^13^ in a flexible way and at a high repetition rate, however these also present significant gradients. Another method, which we have investigated, is to reuse the concept of a hohlraum, i.e. a cylinder, but this time filled with a low-density foam instead of a gas, and ionizing the foam with an external radiation source. The main motivation for using a cylinder is first to allow handling of the low-density foam which would otherwise be extremely difficult, and also to confine the plasma produced by the foam ionization, helping to maintain its density homogeneity. Such target was for example used in ref. 12 where cylindrical irradiation of the cylinder by high-power lasers was used to ionize the foam. We have also used the same target concept where longitudinal ionization of the foam was performed to produce the plasma medium used for laser propagation studies^14^.

In this paper, we report on the characterization (density, temperature, and uniformity in 2-D), as achieved by x-ray^15^ and proton^16^,^17^ radiography, of the plasma that is contained within the cylinder and that was used in the study of ref. 14. The plasma that is heated and confined in the cylinder has temperatures of 5–10 eV and electron density around 10^21^ cm^−3^ (which is tunable by adjusting the density of the foam filling the cylinder). This well-characterized platform of uniform density and temperature plasma is of interest to perform investigations dedicated to High-Energy Density Physics using large-scale laser platforms^18^ where few shots are available.

## Experimental Setup

**Figure 1:**
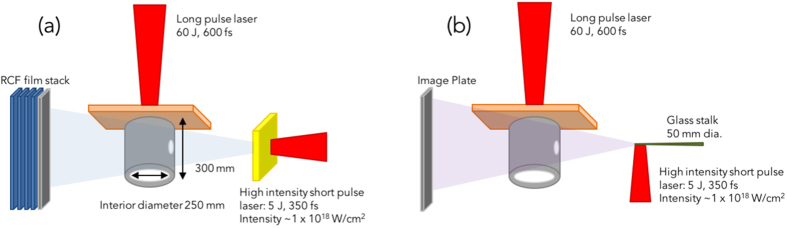
Experimental setup of the plasma creation and diagnosis with (**a**) proton radiography and (**b**) x-ray radiography.

The experiment was performed at the ELFIE laser facility at LULI, Ecole Polytechnique, France, where both short-pulse and long-pulse laser beams are available^19^, using the experimental setup shown in Fig. 1. The target consisted of a polyimide tube, filled with low density foam in the interior, and had a thin (0.7 μm thick) Cu foil at one end of the tube. The length of the tube was 300 μm, and the inside diameter of the tube 254 μm. The plastic (C_15_H_20_O_6_) foam was filled in the tube with a density of 5 mg/cm^3^ (±5%), which corresponds to a particle density of 3.4 × 10^20^ atoms.cm^−3^, or 20 mg/cm^3^ (±5%), which corresponds to 1.37 × 10^21^ atoms/cm^3^. Also shown in Fig. 1, are two holes (with diameter 75 μm) that were laser-drilled on the tube wall to allow a free line of sight directly to the foam for the diagnostics so that they are not hindered by the tube walls.

To fill the hollow tubes with the foam, a series of delicate operations need to be performed. First, an adequate acrylate monomer/mixture of monomers was dissolved in a suitable solvent and injected inside the tube using a micro needle. The solution was then illuminated with UV light for curing. The solution inside the target cavity gels within seconds (*in-situ* polymerisation). The target containing the wet foam is transferred to a critical point dryer, and using liquid CO_2_ as critical solvent the wet gels are dried.

The 700 nm thick Cu foil was attached to one side of the tube to act as the converted material from laser light to a burst of x-rays in order to ionize the foam. The x-rays are created by irradiating the Cu foil with an infrared (1.057 μm wavelength) long-pulse laser pulse (60 J/600 ps) with a focused intensity of 10^14^ W/cm^2^ (using a random phase plate^20^ to homogenise the laser intensity distribution). The use of a phase plate has been shown to aid in conversion of a irregular foam material into a uniform plasma^21^.

The plasma produced by the ionization of the foam was then diagnosed using two methods: proton radiography and x-ray radiography. X-ray radiography is nowadays a widely used technique used in probing dense media. The setup for the creation of the x-ray source is illustrated in Fig. 1(a), where a high intensity short pulse laser irradiated side-on a glass stalk. The interaction of the laser pulse with the plasma generated on the surface of the stalk produces relativistic electrons. Since their mean free path is large (~1 mm), they in turn can induce K-alpha line emission from the non-ionized part of the stalk, far away from the laser interaction region, which avoids shifting the K-alpha line due to plasma effects. Other factors in choosing the laser intensity include: high enough laser intensity to have enough hot electrons (and hence enough x-rays), but it cannot be too low otherwise the laser-to-electrons conversion efficiency drops^22^. As a result, the intensity of the laser was adjusted to 10^18^ W/cm^2^. Since the stalk in perpendicular to the tube axis, seen from the tube, the x-rays are emitted from a micron sized source. The x-rays, having a duration on the order of the laser pulse, can therefore be used to backlight a hydrodynamically evolving (i.e. over ns time scale) dense object in a snapshot. The radiograph is then recorded on x-ray sensitive material; in our case, we used an imaging plate^23^, although active sensor (CCD, MCP, etc.) could also have been used.

Proton radiography is another technique used for probing dense media; the picosecond duration proton burst is here created (see Fig. 1(b)) with the same short pulse laser used for the x-ray radiography detailed above. This technique produces a broadband, laminar proton beam^24^ distributed in a cone having an aperture ~20° (variable depending on the proton energy)^25^. The duration of the proton beam is of several picoseconds, again ideal for probing a hydrodynamically changing medium; and its source size is a few microns, hence allowing extremely good spatial resolution of a probed object. As shown in Fig. 1(b), the proton beam was created here by having the high intensity short pulse laser irradiate face-on an Au foil. This proton beam is sent through the target and a projected 2-D image is recorded in a stack of radiochromic films (RCF) sensitive to protons^25^. Each RCF film detects a narrow energy range of the incident protons (ΔE  ≃ 1 MeV) due to the Bragg peak associated with the energy deposition of the protons in matter. As a result of the difference in time of flight of protons within this 1 MeV energy range, the temporal integration through the tube structure of the probing protons in each film corresponds to ≃20 ps, i.e. much shorter than the hydrodynamic plasma evolution timescale. The probing proton beam is sensitive to many plasma parameters, but most sensitive to electric and magnetic fields (which can also be diagnosed this way) and the density of the medium^16^, which makes this technique well suited for plasma density verification. The density of a compressed plasma can be retrieved either through the scattering^16^,^26^ or the induced spectral shift it induces on the probing protons. However, since the foam is here of low density, the proton beam cannot be used to determine with good accuracy the density of the foam as the protons are of too high energy for this purpose, whichever technique is used. Nonetheless, the protons can be used to track density changes such as shockwaves, which are detectable due to their associated higher density (see below).

## Simulations of the Foam Ionization

**Figure 2:**
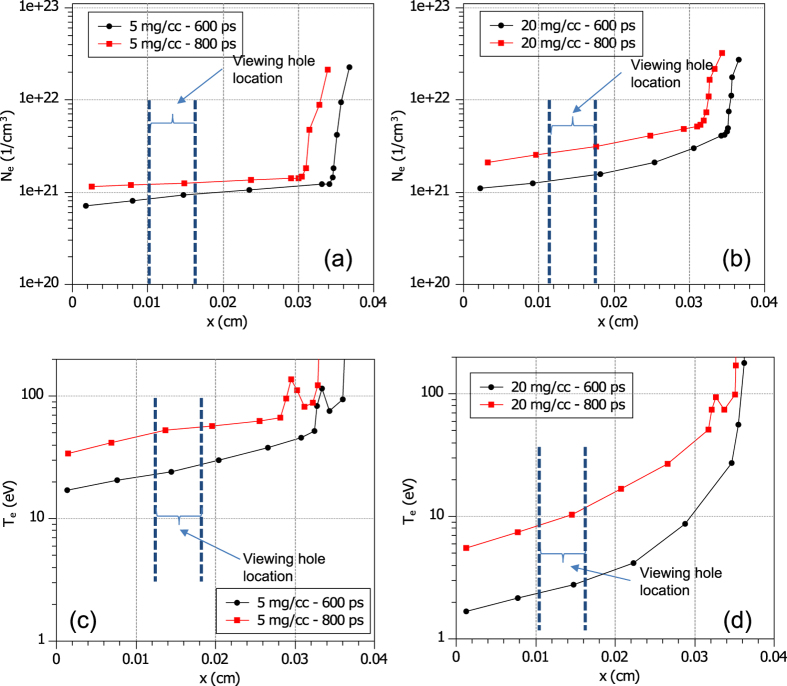
Results of the x-ray heated foam from 1-D simulations performed using the code CHIC. The laser came from the right side and irradiated a copper foil to convert the energy into x-rays. Two foam densities were used in the simulations: 5 and 20 mg.cm^−3^. For two snapshots in time, 600 and 800 ps after the beginning of the laser irradiation of the x-ray converter (the Cu foil), the density and temperature of the heated foam is shown.

We have performed 1D hydro-radiative simulations with the code CHIC^27^ to verify what the plasma parameters would be from the ionization of the foam by the x-ray produced from the copper target and to see the influence of different parameters of the laser used to irradiate the Cu foil. Figure 2 shows the results concerning the density and the temperature at 600 and 800 ps following the beginning of the long-pulse laser irradiation of the Cu foil. In our simulations, the long-laser pulse rises linearly over 100 ps to a peak intensity of 1 × 10^14^ W/cm^2^, and has a duration of one nanosecond. We assume that the foam structure, consisting of micron size voids with irregularly shaped surrounding walls converts instantaneously to a uniform plasma. Consequently, a uniform and homogeneous medium at equivalent density (C_15_H_20_O_6_) is used in computations. In each plot in Fig. 2, the laser comes from the right side. The foam, having density 5 or 20 mg.cm^−3^, is 400 μm long. From the results of the simulations (see in particular Fig. 2(c,d)), we can see the propagation of the electron temperature front into the foam and a shock has begun to form. The electron temperature increases with time and reaches to Te = 20–40 eV for the 5 mg.cm^−3^ case and Te = 2–10 eV for the 20 mg.cm^−3^ case after a few hundreds of ps. At 600 ps, the average degree of ionization was Z = 2.1 (T_e_ ~ 20 eV) for the 5 mg.cm^−3^ foam and Z = 0.7 (Te ~ 3 eV) for the 20 mg.cm^−3^ foam. The electrons density had then reached several times the critical density, which was the aim of the target design and is extremely uniform through most of the tube. Near the copper converter foil, the temperature and density are higher, and a shock has formed. On the other end of the tube, the foam heating induces a small plasma expansion and consequently a small density gradient at the end of the foam which could reach a few microns. The viewing hole for the diagnostics is centered at 150 μm from the end of the tube (positioned at x = 0 in the simulation results).

## Results and Discussion

### Point projection X-ray radiography

X-ray radiography has been successfully used in the past to determine plasma density. Here, we used a similar technique to measure the density profile of the foam inside the cylinder. As mentioned above, the x-ray source was created by shooting a glass stalk having a diameter of 50 μm by a short pulse laser to create the 1.74 keV K-alpha line emission. The spatial uniformity of the backlighting x-rays was measured to be ±0.3% across 500 μm at the object plane (from it, we derive the uncertainty in the transmission measurements shown in Fig. 4), and the spatial resolution is better than 20 μm. The backlighter short pulse beam was triggered 600 ps after the beginning of the long pulse beam heating the foam. The radiograph was recorded on an imaging plate (FujiFilm TR) with 13 μm thick black Kapton in front of it acting as a light-tight filter. As shown in Fig. 1(a), we do not use an imaging system, but rather simply have a projection of the target on the imaging plate. In this projection scheme, the target is magnified by a factor 10 on the detector.

**Figure 3:**
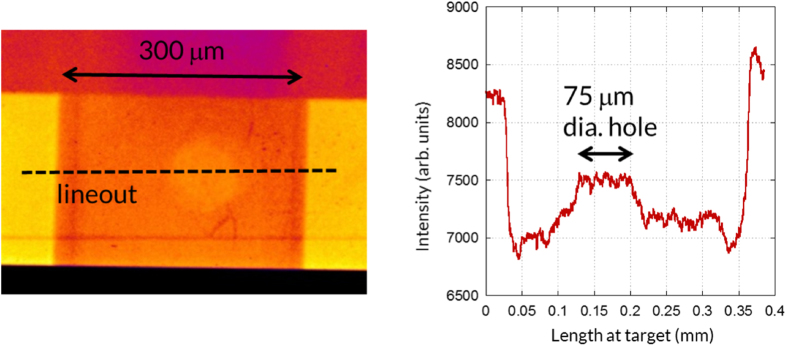
(left) A sample x-ray radiograph of the tube and 20 mg/cm^3^ density foam, which was here ionized by the x-rays produced by the long-pulse laser, as recorded on the imaging plate. (right) A lineout was taken across the aligned holes (and along the dashed line shown on the image). It is used to determine the transmission through the foam. Note that the variation of the x-signal across the hole is of the order of 0.7%, and 0.3% of the background x-ray signal over the large area used for deducing the transmission.

To verify the x-ray spectrum originating from the stalk, we used a series of step filters laid on the imaging plate surface. By measuring the transmission of the signal through these filters, and by adjusting the incident spectrum to fit these recorded transmissions, that spectrum can be inferred. Such process of the reconstruction of the spectrum from the step filters showed that the cold K-alpha line is more than a hundred times brighter than the background (Bremsstrahlung), which is sufficient to assume that the energy deposited on the imaging plate is predominantly the K-alpha x-ray^28^. A sample image, of a target where the foam was ionized, from the experiment is shown in Fig. 3(a). A lineout, plotted in Fig. 3(b), was taken at the center of the view hole and the average value of the transmission through the foam (indicated in Fig. 3(a,b)) was used in the analysis.

**Figure 4:**
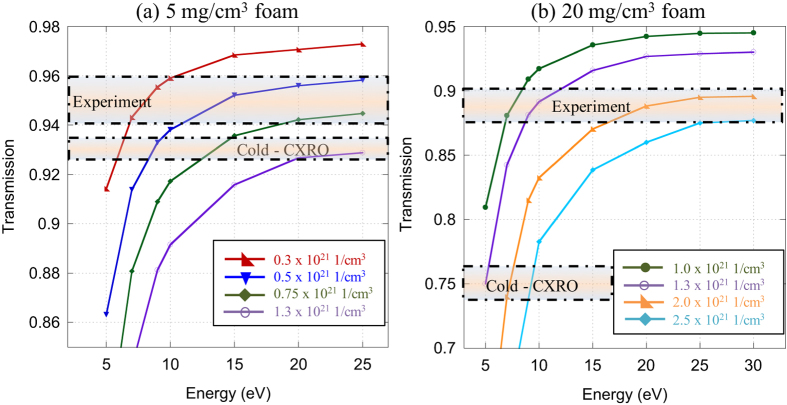
Simulation results from the atomic code FLYCHK. (**a**) The cold transmission of a 5 mg.cm^−3^, 250 μm long foam is 93% to 1.7 keV x-rays. The heated foam showed a transmission of 95%. The colored curves are simulated transmissions from FLYCHK for four different densities. (**b**) The cold transmission of a 20 mg.cm^−3^, 250 μm long foam is 75% to 1.7 keV x-rays. The heated foam showed a transmission of 89%. The colored curves are simulated transmission from FLYCHK for four different densities.

We present here the analysis of plasma density and temperature retrieved for targets having the two foam densities. Figure 4(a) presents the calculations and data used in this procedure for the target that contained a 5 mg.cm^−3^ density foam. First, using the database of CXRO^29^, we calculated the transmission the foam would have before ionization. This calculation is done based on the cold (5 ± 0.25) mg.cm^−3^ density of the foam, yielding a transmission of (93 +- 0.4)%. In the experiment, we measured a transmission of (95 ± 1)% through the ionized foam, thus indicating a change in density and temperature. For the target that contained a foam with 20 mg.cm^−3^ density, the results are illustrated in Fig. 4(b). The cold transmission for this target as calculated by CXRO is (75 +- 1.5)%. We measured a transmission of (89 ± 1)% through the ionized foam during the experiment.

Then, we used the collisional-radiative atomic code FLYCHK to calculate the transmission, for different temperatures and densities, of a partially ionized C_15_H_20_O_6_ foam. Since FLYCHK does not have the option of working with complex compounds, the opacity of each element was calculated in each condition and a weighted summed was made. The thickness of the foam used in the calculation was 250 μm, i.e. as in the experiment. For the plasma densities used in the FLYCHK calculations, we used the density predicted by CHIC (see Fig. 2(a,b)), plus two additional densities around that density, at the time of the x-ray probing and at the location of the hole in the tube. For a given density, we varied the plasma temperature from 5 to 20 eV, which resulted in different plasma ionization. FLYCHK then gives the predicted x-ray transmission through the chosen material. This is presented in Fig. 4 for both the 5 mg.cm^−3^ and 20 mg.cm^−3^ foam.

We can see in Fig. 4(a) that for the foam 5 mg.cm^−3^, the measured transmission can correspond, given the uncertainty on the transmission, to a range of plasma conditions. From the results of the CHIC simulations, for the same 5 mg.cm^−3^ foam, we were expecting at the time and location of the x-ray probing the foam to have a temperature of 20 eV and an ionization state of 2.1, which would give an electron density of 8 × 10^20^ cm^−3^. Hence, the set of plasma conditions as input to FLYCHK of 0.75 × 10^21^ cm^−3^, ~16 eV, gives an x-ray transmission that we observed in the experiment. As for the 20 mg.cm^−3^ foam, CHIC predicts a temperature of 2.2 eV and an ionization state of 0.7, which would give an electron density of 1.1 × 10^21^ cm^−3^. Then, looking at Fig. 4(b), we can see that the set of plasma parameters of 1 × 10^21^–3 × 10^21^ cm^−3^ and 7 eV would be best at matching the experimental data. Here, the temperature predicted by CHIC is somewhat higher than found from the experimental data, but still of the same order of magnitude. It should be noted that if the x-ray backlighter was shifted to higher energy by ionization to He-like Si at 1.85 keV, it would change the transmissions curves shown in Fig. 4 by about 1%.

### Proton radiography

As mentioned above, we probed the targets with high energy protons, mostly to verify the homogeneity of the produced ionized plasma. The radiograph was recorded using a stack of radiochromic films (Gafchromic HD-810) with a 10 μm thick aluminum foil in the front as light-tight filter. Short pulse laser produced proton beams have picosecond time-scale bunches, hence are sufficiently short to probe nanosecond time-scale phenomena. The high intensity short pulse laser irradiated a 10 μm Au foil (see Fig. 1(b)), and by the TNSA acceleration process^24^,^25^, a broadband proton beam with a cutoff of around 10 MeV was produced.

**Figure 5:**
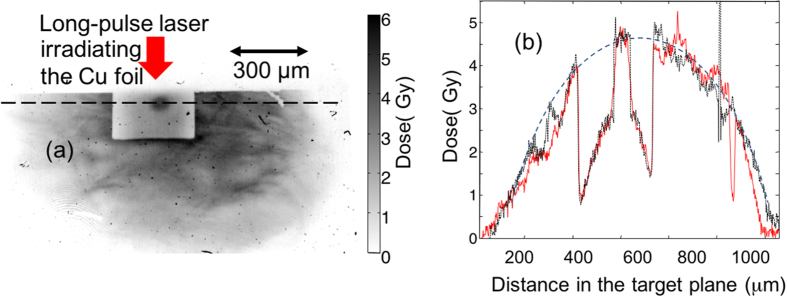
(**a**) A sample image of a proton radiograph at 6 MeV of the ionized foam (density 5 mg.cm^−3^) and tube. (**b**) Line out through the hole (i.e. along the dashed line marked in (**a**)), showing the proton dose beam across the tube and through the hole. In red (full line) is shown the raw lineout. In black (dotted) is shown the lineout deconvolved from the scattering at the edges of the hole. The scattering in the low-density foam itself (of the order of 0.15° 30) is negligible. In grey (dashed) is shown the interpolation (following an ellipse shape) of the incident dose based on the dose apparent on both sides of the tube. One can see that the interpolated dose at the location of the hole corresponds quite well to the dose recorded there.

Figure 5 shows an example of proton radiography obtained during the campaign, as detected on RCF. The probing is performed at the same time as the x-ray probing shown in Fig. 1. The long pulse heating beam was incident from the top of the image. One can clearly see the hole in the tube for probing.

The protons produced by the high-intensity auxiliary laser have typically an exponentially decreasing spectrum^25^. When probing a dense medium, one way to diagnose the density of the medium is to take advantage of the slowing down of the protons in the medium which will shift the exponential spectrum to lower energies. By comparing the incident spectrum of the protons to the one recorded through the probed object (the technique to retrieve the proton spectrum from the stack of RCF is detailed in ref. 30), one can thus measure the integrated density along the proton line-of-sight. Here however, this method cannot be used since the medium (the ionized foam) is of low-density and the protons are of high energy (≥1 MeV). Indeed, for such high-energy protons, the shift in energy in the foam would be of the order to 40 keV, hence modifying the spectrum too little for the shift to be detected. In fact, as can be quantitatively seen in Fig. 5(b), the dose recorded through the foam (as seen through the hole) is indeed the same as the incident dose of protons, consistent with very little shift of the spectrum.

Nonetheless, we can observe that the projected image of the protons through the diagnostic hole is very smooth and uniform. This is consistent with the CHIC simulations shown in Fig. 2 which indicates that the plasma ionization at the time of protons generates a uniform medium. In particular, we can assess that the shock front has not reached the observation window (the hole), otherwise the density jump associated with it would be detectable (not through the induced scattering, which would be too low [~0.6°], but through the induced spectral shift of ~0.6 MeV, which would induce, for our retrieved spectrum, a change of dose at 6 MeV of ~8.5% when the uncertainty on the retrieved dose at 6 MeV is ~3% and the fluctuations in the dose observed in the hole (see Fig. 5(b) are ~4%. However, smaller density variations (e.g. by a factor ~2–3) would not be detectable.

## Conclusion

We have tested and characterized a platform for generating homogeneous, well-controlled, long scale-length plasmas of density close to the critical density. This platform is composed of a tube (to constrain the plasma expansion) filled by a low-density foam. At one end of the tube, a thin Cu foil is positioned; it is irradiated by a long-pulse laser that generated x-rays ionizing the foam. 1-D hydrodynamic-radiative simulations suggest that the ionization produces a rather uniform plasma in the tube, at temperatures of a few eVs. X-ray probing allows us to retrieve the density and temperature of the ionized foam. Proton probing shows additionally that the produced plasma is uniform and void of shock waves. This platform of controllable density (by varying the initial density of the foam) and temperature (by varying the flux of the x-rays ionizing the foam) is of interest for performing measurement requiring such plasma, i.e. laser propagation, or measurement of ion stopping in dense, hot plasma.

## Additional Information

**How to cite this article**: Chen, S. N. *et al.* Density and temperature characterization of long-scale length, near-critical density controlled plasma produced from ultra-low density plastic foam. *Sci. Rep.* **6**, 21495; doi: 10.1038/srep21495 (2016).
